# Novel Cast Polyurethanes Obtained by Using Reactive Phosphorus-Containing Polyol: Synthesis, Thermal Analysis and Combustion Behaviors

**DOI:** 10.3390/ma14112699

**Published:** 2021-05-21

**Authors:** Izabela Zagożdżon, Paulina Parcheta, Janusz Datta

**Affiliations:** Department of Polymer Technology, Faculty of Chemistry, Gdansk University of Technology, G. Narutowicza St. 11/12, 80-233 Gdańsk, Poland; izabela.zagozdzon@pg.edu.pl (I.Z.); paulina.parcheta@pg.edu.pl (P.P.)

**Keywords:** phosphorus-containing polyol, cast polyurethanes, flame retardants, thermal properties, combustion behaviors.

## Abstract

Phosphorus-containing polyol applications in polyurethane synthesis can prevent volatilization of flame retardants and their migration on the surface of a material. In this work, novel cast polyurethanes were prepared by a one-step method with the use of different amounts of phosphorus-containing polyol, 4,4′–diphenylmethane diisocyanate and 1,4-butanediol. The chemical structure, thermal, physicochemical and mechanical properties and flame resistance of the prepared materials were investigated. The results obtained for cast flame-retarded polyurethanes were compared with cast polyurethane synthesized with commonly known polyether polyol. It has been shown that with an increasing amount of phosphorus content to polyurethane’s chemical structure, an increased flame resistance and char yield were found during combustion tests. Phosphorus polyol worked in both the condensed (reduced heat and mass exchange) and gas phase (inhibition of flame propagation during burning). The obtained materials contained phosphorus polyol, indicating higher thermal stability in an oxidative environment than an inert atmosphere.

## 1. Introduction

Cast polyurethanes are characterized by good physical and chemical properties and also high durability; therefore, they are commonly known in the plastic industry. They are often used to produce suspension bushes, boat trailer rollers, seals for pumps, cyclones liners and spigots, conveyor belts or encapsulation electrical and electronic elements. Their properties depend on the type of used components, chemical structure, average molecular weight, method of polymerization and the molar ratio of isocyanate groups to hydroxyl groups [NCO]/[OH] [1,2,3]. However, polyurethanes (PUs) have disadvantages, with the main one being high flammability. Therefore, the use of appropriate flame retardants is important to reduce the burning rate and examine the combustion behaviors of these materials [4,5].

The main components used in cast polyurethane production are polyols, isocyanates and low molecular weight chain extenders. Polyols are dominant components that can pose even more than 60 wt.% of PUs [1,6]. Due to the high flammability of polyurethanes, in order to increase their usage security, the combustion inhibitors are used. They can be divided into two groups: additive and reactive flame retardants (FRs). The first are added to the polymer during its processing. This type often require the use of additional flame retardants in order to achieve a synergistic effect. The reactive flame retardants form compounds containing functional groups, which can be chemically incorporated into the polymer chain during synthesis. This method prevents volatilization or migration of the combustion inhibitor to the surface of the material [7,8]. The most popular and promising substances used in recent years to obtain polyurethanes with improved thermal stability and flame retardancy are metal compounds [5,9,10], melamine and its derivatives [11], boron compounds [12], polyhedral oligomeric silsesquioxanes [13], functionalized graphene oxide [14,15], expanded graphite [16], carbon nanotubes [17] and phosphorus-containing substances. Due to environmental and health aspects, some halogenated substances, e.g., brominated and chlorinated compounds, are excluded from use, despite their high effectiveness [18,19].

Phosphorus flame retardants work in the condensed or the gas phase. They contribute to the formation of a char on the surface of the solid phase, which limits further access of heat and air to the burning material. They also prevent entering material decomposition gas products into a flame zone. As a result of the thermal decomposition of phosphorus compounds, phosphoric acid is mostly formed, which condenses quickly to obtain pyrophosphate structures (P-O-P) and H_2_O. Phosphorus protects the polymer and the water rarefies the oxidizing gas phase. Phosphorus-containing combustion inhibitors can also take on the role of free radical scavengers in a gas phase. When they are incorporated into the polymer structure, they play an effective role in reducing heat release [18,20].

Phosphorous-based flame retardants comprise a wide range of products. Among them are red phosphorus (RP), inorganic phosphates and organic–inorganic compounds containing incorporated phosphorus atoms. However, red phosphorous has a major disadvantage, which is the release of highly toxic phosphine during melting and requires microencapsulation (mRP) [18,20]. Regarding ammonium polyphosphate (APP), there are some problems with its practical use. It is characterized by low flame retardancy, which makes it necessary to use it in large amounts. The disadvantage of using APP is the need to add it to PU together with a synergistic flame retardant [21].

Organophosphorus monomers are incorporated directly into the polyurethane structure. A. H. Yang et al. [22] obtained a polyphosphate ester containing Shiff-bases (SPE) and used it for the synthesis of thermoplastic polyurethane elastomers. It was shown that, with increasing amount of SPE, the content of residue at 700 °C in the thermogravimetric analysis also increased. The elastomer with the highest SPE content was classified as V-0 during the UL-94 vertical test, the LOI value was 29% and the heat release rate decreased by 61.7% compared to materials without SPE. W. H. Rao et al. [23] obtained phosphorus polyol (PDEO) by the reaction of phenylphosphonic dichloride and with ethylene glycol. Synthesized PDEO was used for the preparation of polyurethane materials. The incorporation of PDEO into the polymer matrix resulted in increased thermal stability and improved fire resistance. The residues at 600 °C in the TGA and LOI tests increased with the higher PDEO content in PU. H. Chen et al. [24] prepared a series of waterborne polyurethanes with different contents of reactive organophosphate Exolit^®^ OP 550. The obtained materials showed significantly higher char yields compared to the material without Exolit^®^ OP 550. Although the mechanical properties worsened, the combustion behaviors greatly improved—the LOI increased to 32% at 10 wt% organophosphate content.

The aim of this work was to investigate the novel cast polyurethanes characterized by improved flame retardancy and thermal stability, which could be used to encapsulate electrical and electronic elements. The obtained materials were synthesized by using different amounts of reactive phosphorus-containing polyol via a one-step polymerization method. The combustion behaviors were measured by a cone calorimeter, limiting oxygen index (LOI) method and the vertical burning test (UL-94). Thermal properties were performed with thermogravimetric analysis (TGA), both in nitrogen and air atmospheres, and dynamic mechanical thermal analysis (DMTA) measurements. Moreover, the chemical structure and physicochemical and mechanical properties were also investigated.

## 2. Materials and Methods

### 2.1. Materials

Polytetrahydrofurane (PTHF) (molecular weight: 1000 g/mol, hydroxyl number: 106.9–118.1 mg KOH/g, functionality: 2) was obtained from Cortex Chemicals (Tarnów, Poland). Highly reactive, halogen-free polyol, containing built-in phosphorus atoms, Exolit^®^ OP 550 (OP) (molecular weight: 680 g/mol, hydroxyl number: 170 mg KOH/g, phosphorus content: 16.0–18.0% w/w, functionality: 2), was kindly supplied by Clariant (Frankfurt, Germany). 4,4′–Diphenylmethanediisocyanate (MDI) was purchased from BorsodChem (Bolyai, Hungary) and 1,4–butanediol (BDO) was obtained from Sigma Aldrich (St. Louis, MO, USA).

### 2.2. Synthesis of Polyurethanes

Before the syntheses, PTHF, phosphorus polyol (OP) and low molecular chain extender (BDO) were dried under vacuum for 2 h at a temperature of 95–100 °C.

The reference polyurethane was synthesized by the polyaddition reaction of dried polytetrahydrofurane (30.46 g) with 4,4′–diphenylmethane diisocyanate (20.04 g) and dried 1,4-butanediol (4.29 g). All monomers were mixed at 70 °C for 1.5 min under vacuum. After degassing, the mixture was poured into a preheated mold (80 °C).

**Figure 1:**
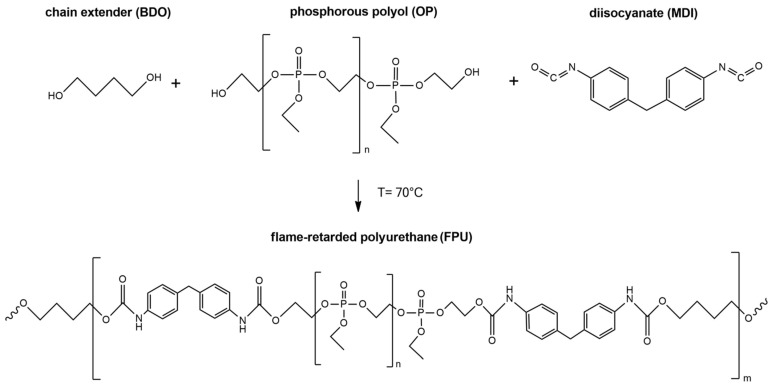
Scheme of the synthesis of flame-retarded cast polyurethanes by one-step method.

Polyurethanes, which contain incorporated phosphorus atoms, were synthesized by the method described in patent application no. P.434425 (Polish Patent Office). All substrates used for the synthesis were heated to 70 °C. For preparation of material coded FPU-8.5, firstly, dried OP (27.84 g) was mixed with MDI (22.66 g) and dried BDO (4.29 g) under vacuum at a temperature of 70 °C. The reaction was carried out until the viscosity increased visibly (max. 6 min). After mixing and degassing, the mixture was poured into a preheated mold (80 °C). Figure 1 shows the scheme of the synthesis of new flame-retarded cast polyurethanes.

All cast PUs was cured at 80 °C for 24 h in a laboratory oven to complete the reaction between [NCO] and [OH] groups. The molar ratio of [NCO]/[OH] groups equaled 1.0 for all polyurethane materials. The flame-retarded polyurethanes differed in the molar ratios of substrates and percentage contents of phosphorous atoms (FPU-8.5, FPU-9.0 and FPU-9.5). The reference material was labeled as REF and it did not have any incorporated phosphorus atoms. Formulations of materials and a quantity of phosphorus in PUs are shown in Table 1.

### 2.3. Methods

The Fourier transform infrared spectra (FTIR) were acquired with the use of Nicolet FTIR 8700 spectrophotometer (Thermo Electron Corporation). The wavenumber ranged from 500 to 4000 cm^−1^ and the spectra were registered at room temperature with a resolution of 4 cm^−1^ and 64 scans. A carbonyl hydrogen bonding index (R), a degree of phase separation (DPS) and a degree of phase mixing (DPM) have been calculated according to the formulas:

(1) $$R=\frac{A_{z}}{A_{w}}$$

(2) $$\mathrm{DPS}=\frac{R}{R+1}$$

(3) DPM=1−DPS

where A_z_ is an absorbance value of hydrogen-bonded carbonyl groups at the urethane bonds and A_w_ is the absorbance value of free carbonyl groups from the urethane bonds [1,25].

Hard (HS) and soft segment (SS) contents have been calculated according to the formulas:

(4) $$\mathrm{HS}=\frac{m_{\mathrm{MDI}}+m_{\mathrm{BDO}}}{m_{\mathrm{MDI}}+m_{\mathrm{BDO}}+m_{\mathrm{OP}}}\cdot 100\%$$

(5) $$\mathrm{SS}=\frac{m_{\mathrm{OP}}}{m_{\mathrm{MDI}}+m_{\mathrm{BDO}}+m_{\mathrm{OP}}}\cdot 100\%$$

where m_MDI_, m_BDO_ and m_OP_ are mass of MDI, BDO, OP [g], respectively [26].

X-ray diffraction (XRD) was used in order to determine the structural properties of the obtained flame-retarded cast polyurethanes. The measurements were conducted with a Phillips X’Pert Pro diffractometer (XRD) with CuKα radiation of wavelength λ = 1.540 Å in 2θ angel in the range from 10 to 90° under 40 kV and 30 mA, at room temperature in air atmosphere.

Dynamic mechanical thermal analysis (DMTA) was conducted using a DMA Q800 Analyzer (TA Instruments) in accordance with the ISO 6721-1:2019 standard [27]. The 40 mm × 10 mm × 2 mm stripe-shaped samples were thermostated at −100 °C for 1 min, then heated from −100 °C to 150 °C with a rate of 4 °C/min.

Thermogravimetric analysis (TGA) was carried out using a NETZSCH TG 209F3 analyzer. The weight of each sample was ca. 10 mg. The measurement temperature ranged from 35 to 700 °C, at a rate of 10 °C/min. The analyses have been conducted both in nitrogen and air atmospheres.

Vertical burning test (UL-94) was performed in accordance with the PN-EN 60695-11-10:2014-02 standard [28]. The material samples with dimensions of 127 mm × 12.7 mm × 2 mm were placed vertically in the holder. Then, they were exposed to the fire at an angle of approximately 45° for 10 s and the fire was removed. When burning was stopped, the sample was exposed to the fire for an additional 10 s.

Limited oxygen index (LOI) values were measured using Concept Equipment oxygen index meter on stripe-shaped samples of size 100 mm × 10 mm × 4 mm according to the PN-EN ISO 4589-2:2017-06 standard [29].

Cone calorimetry (CC) was conducted using iCone mini calorimeter by Fire Testing Technology according to ISO 5660-1:2015 [30] under heat flux of 50 kW/m^2^. The dimension of specimens was 100 mm × 100 mm × 4 mm. A flame spread rate (FSR) has been calculated according to the formula:

(6) $$\mathrm{FSR}=\frac{\mathrm{pHRR}}{\mathrm{TTI}}\left(\frac{\mathrm{kW}}{m^{2}\cdot s}\right)$$

where pHRR is a maximum heat release rate (kW/m^2^) and TTI (s) is a time to ignition [31].

Tensile properties were carried out using a Zwick/Roell Z020 universal testing machine according to the PN-EN ISO 527-1:2020-01 standard [32]. The tensile tests were performed at room temperature. The paddle-shaped samples were tested at a rate of 100 mm/min.

Hardness was measured in accordance with the PN-EN ISO 868:2005 standard [33] by using a Zwick/Roell Shore durometer, type D. All measurements were carried out at room temperature. The presented results are the mean values of hardness based on ten independent measurements.

Density of materials was determined by using a RADWAG electronic analytical balance, in accordance with PN-EN ISO 1183-1:2019-05 standard [34]. The measurements were performed at room temperature with the use of methanol as an immersion liquid.

## 3. Results and Discussion

### 3.1. Fourier Transform Infrared Spectroscopy Results (FTIR)

**Figure 2:**
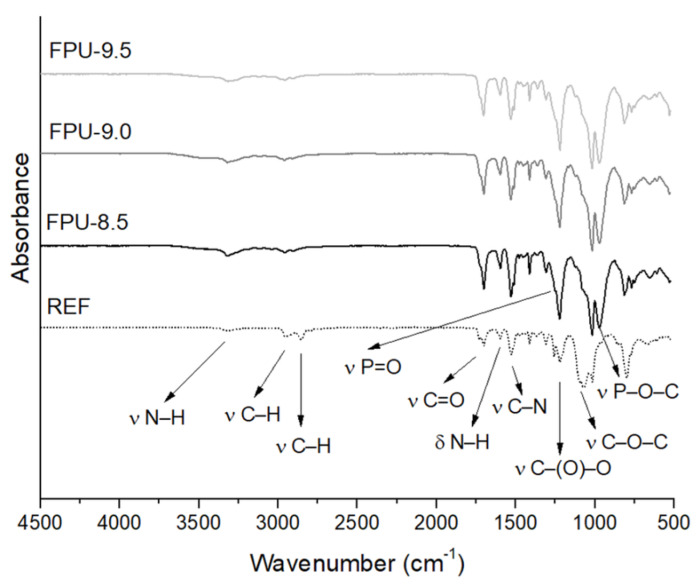
FTIR spectra of polyurethanes containing different amounts of phosphorus polyol (FPU-9.5, FPU-9.0, FPU-8.5) and reference material (REF).

Figure 2 shows the FTIR spectra of all synthesized materials. The vibrations derived from characteristic functional groups have been analyzed. N–H stretching vibrations derived from the urethane group were registered at the wavenumber 3320 cm^−1^. The peak at 2940 cm^−1^ corresponds to asymmetric vibrations of the –CH_2_ groups, while the symmetrical vibration of the –CH_2_ groups occurred at 2855 cm^−1^. The lack of a visible peak at 2270 cm^−1^ confirmed the almost complete conversion of isocyanate groups to urethane groups. Depending on the origin, the peak indicating the symmetric vibration of a carbonyl groups could occur in the range 1670–1750 cm^−1^ [35]. The multiplet band of stretching vibrations of the C=O group, derived from the urethane group, appeared at 1730 cm^−1^ for free carbonyl groups, when at 1700 cm^−1^ the band of the hydrogen-bonded carbonyl groups is visible. The peak assigned at 1597 cm^−1^ was attributable to the N–H bending vibrations. The stretching vibrations of C–N bonding occurred at 1529 cm^−1^. At 1414 cm^−1^, the bending vibrations of the methylene groups are visible. The band presented at 1223 cm^−1^ was derived from stretching vibrations of the carbamate groups –C(O)O. The peaks of materials containing OP (FPU-8.5, FPU-9.0 and FPU-9.5) showed, at this wavenumber, higher absorbance values—in this instance, the band derived from the stretching vibrations of –P=O bond was overlapped to the peak of carbamate groups. Bands at 1088 cm^−1^ corresponded to the stretching vibrations of the ether groups in the REF material. Stretching vibrations at 1017 and 979 cm^−1^ confirmed the presence of P–O–C groups in OP-containing polyurethanes. Stretching vibrations of –CH groups derived from the aromatic ring are visible at 800 cm^−1^. Spectra of materials containing OP confirm the incorporation of phosphorus polyol into the chain of obtained polyurethanes [24,35,36,37].

Table 2 contains the values of hydrogen bond index (R), degree of phase separation (DPS), degree of phase mixing (DPM) after the deconvolution of spectrum in the carbonyl regions and the hard (HS) and soft (SS) segment contents in the obtained PUs. The hard segments of polyurethane are derived from isocyanate and low molecular weight chain extender, while the soft segments are derived from polyol [26]. The presented results confirmed the degree of phase separation in the range of 0.60–0.70 for all tested samples of PU materials. A greater content of hydrogen bonds between urethane groups can be obtained due to a higher amount of diisocyanate [25]. The highest degree of phase separation was revealed in FPU-8.5, which characterized also the highest content of HS (49.19%). The strong hydrogen bond between the hard segments is one of the most important factors in microphase separation [1,25].

For polyurethane materials containing OP, the hydrogen bond index decreases with increasing amount of phosphorus polyol [38]. However, the presence of OP in the PU structure increased the DPS values compared to the REF material. Comparing materials REF and FPU-9.5, which had similar contents of hard and soft segments, due to the lower molecular weight of phosphorous polyol (680 g/mol) compared to PTHF (1000 g/mol) and therefore its shorter chain, there was a greater possibility of more urethane bond formation in OP-containing materials [39].

### 3.2. X-ray Diffraction (XRD)

**Figure 3:**
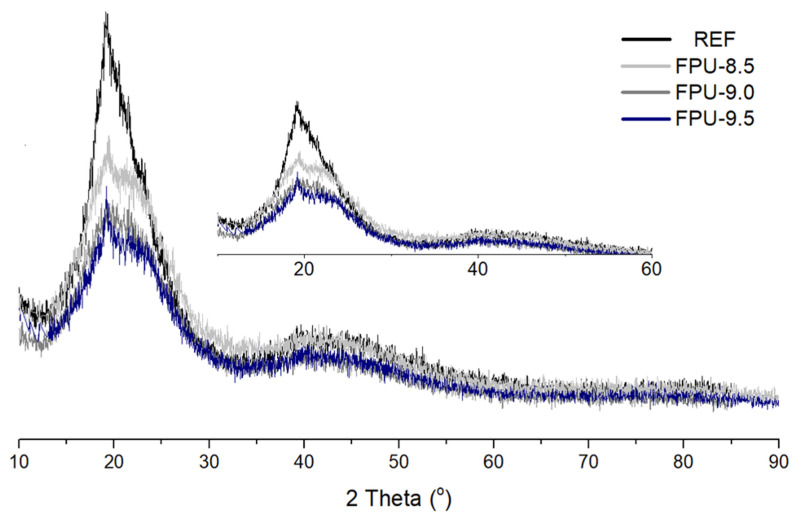
XRD results of the obtained materials.

The XRD results obtained for REF, FPU-8.5, FPU-9.0 and FPU-9.5 materials are presented in Figure 3. All prepared materials exhibit the typical XRD pattern with the most important diffraction signals at 2θ values 20.0, 19.9, 19.7 and 19.5 for REF, FPU-9.5, FPU-9.0 and FPU-8.5, respectively. The most important diffraction signals at 2θ values correspond to the (020) crystal plane [40]. All PUs show a typical crystal phase. Moreover, it was observed that the crystalline structures of the obtained materials decrease with incorporation of phosphorus-containing polyol in the polyurethane structure. The XRD pattern of FPUs show a broad lump between 15 and 25°, which is associated with the increase in the amorphous domain at the polyol crystal structure [41]. The addition of OP could not change the crystalline structure, but could decrease the crystallinity of FPUs structure due to the decrease in the height of the diffraction peaks. We can probably claim that the presence of OP-containing polyol in the macromolecular chain is responsible for disrupting chain regularity and packing in case of polyurethanes [42].

### 3.3. Dynamic Mechanical Thermal Analysis (DMTA)

**Figure 4:**
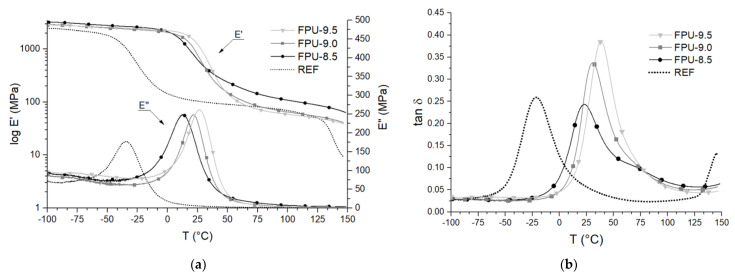
DTMA results: (**a**) the logarithm of storage modulus (log E’) and loss modulus (E”); (**b**) and the damping factor (tan δ) as a function of the temperature (T) of the obtained polyurethanes.

Table 3 and Figure 4 show the results of the dynamic mechanical thermal analysis as a function of the storage modulus (E’), loss modulus (E”) and damping factor (tan δ) versus temperature (T). ∆T is a temperature range of efficient damping (tan δ > 0.3) and S is an integral area in the range of 140 °C in tan δ-temperature curve [43].

The results indicate that, with decreasing percentage of hard segments in the OP-containing polyurethane materials, the value of tan delta increases. The materials with the highest phosphorus polyol contents (FPU-9.0 and FPU-9.5) had tan delta values above 0.3, which proved they good damping properties. For these materials, ∆T was specified, which is defined as the damping temperature range. Tan δ values for REF and FPU-8.5 were similar. S was calculated from tan delta peaks, for FPUs in the range −25 to 115 °C and −70 to 70 °C for REF. This is an additional supplementary indicator to measure damping performance and increased with increasing OP amount. ∆T and S parameters were the highest for polyurethane, which had 9.5 wt% phosphorous atoms content [43].

The presence of OP in the polyurethane structure contributed to an increase in the glass transition temperatures by up to 60 °C, compared to the reference material, the Tg_SS_ value for FPU-9.5 was 38.4 °C when for REF was −21.2 °C. This implies that OP-containing PUs were in a glassy state at room temperature. Obtained materials were plastomers. One of the reasons for this is the low molecular weight of phosphorus-containing polyol [44]. Moreover, the incorporation of an organophosphorus substrate in the polyurethane chain may result in more ordered structures, which led to a limited movement of PU chains and increased the glass transition temperature [45]. The Tg_SS_ values for polyurethanes containing OP were in the range of 25.1–38.4 °C and increased with the amount of phosphorus polyol. Polymers with higher Tg_SS_ values have higher flame retardant activity [4], thus the obtained results could indicate an increase in the fire resistance of materials containing OP compared to REF.

The storage modulus values for PU materials containing OP significantly exceed the value of the reference polyurethane at room temperature (23 °C). These materials were more rigid and higher stresses were needed for their deformation [36]. For REF, the E’_23ᵒC_ was 93 MPa, while for FPU-8.5, FPU-9.0 and FPU-9.5: 719, 927 and 1398 MPa, respectively. The values of E’_23ᵒC_ and E” increased with an increasing amount of phosphorus polyol, despite higher content of soft segments. The presence of the phosphate group could be a kind of a spatial hindrance—the OP chain is less flexible compared to polytetrahydrofurane. Materials with a higher loss modulus irreversibly lose less energy [36].

Implementation of phosphorus polyol in the PU chain improved the dynamic-mechanical properties at the ambient temperature. At higher temperatures, the chains loosen up and the situation was opposite—the E’_SS_ value at 60 °C was the lowest for FPU-9.5 (96 MPa) and the highest for FPU-8.5 (184 MPa).

### 3.4. Thermogravimetric Analysis (TGA)

**Figure 5:**
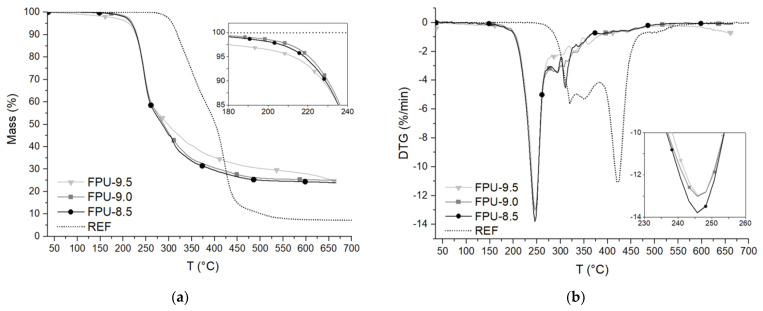

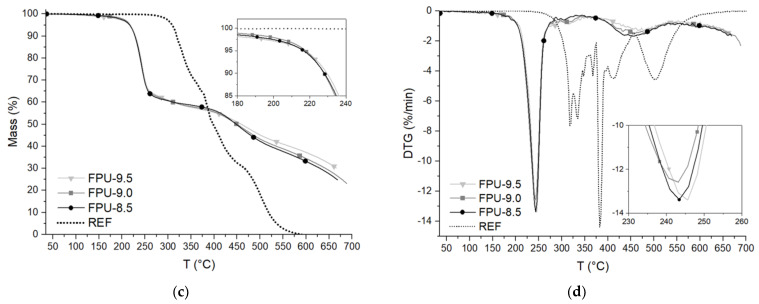
Thermogravimetric analysis results: TGA curves for materials tested in the (**a**) nitrogen and (**c**) air atmospheres and DTG curves for materials tested in the (**b**) nitrogen and (**d**) air atmospheres.

Figure 5 shows the TGA and DTG curves of polyurethane materials containing different amounts of phosphorus polyol or PTHF. The tests were carried out in nitrogen (N_2_) and air (N_2_/O_2_) atmospheres. Table 4 and Table 5 contain a set of obtained results.

The symbols T_5%_, T_50%_ and T_70%_ correspond to temperatures at which the mass loss was 5%, 50% and 70%, respectively. Y is the percentage amount of residual mass of the polyurethane materials after test at 665 °C. Temperatures of maximum rates of mass loss are marked with the symbols T_OP max_, T_HS max_ and T_SS max_ in the nitrogen atmosphere and from T_1 max_ to T_7 max_ in the air atmosphere.

The degradation of the reference material in the nitrogen atmosphere took place in three stages (Figure 5b). The first weight loss peak for the REF was observed at 319.7 °C, and it corresponded to the degradation of hard segments (T_HS max_). The second one, which occurred at 351.7 °C, was probably caused by unreacted polyol residue after one-step method synthesis [46]. The last peak was related to the degradation of soft segments (T_SS max_) and was at a temperature of 422.3 °C.

The decomposition of polyurethanes containing OP in the nitrogen atmosphere consisted of three degradation steps (Figure 5b). The first peak on the DTG curve (T_OP max_) corresponded to the degradation of the phosphate groups. The subsequent peaks at T_HS max_ and T_SS max_ temperatures concerned the degradation of urethane groups in the rigid segments and polyol chains in the soft segments, respectively [47]. This probably resulted from the energy of P–O bond, which is much lower than the C–O bond in the main chain of polyurethane. For P–O, this is 149 kJ/mol and for C–O this is equal to 256 kJ/mol [26,48]. The T_HS max_ and T_SS max_ temperatures of OP-containing PUs were typically much lower compared to the reference material tested in the nitrogen—T_SS max_ differed by up to 100 °C (Table 4). The presence of the phosphoric acid from the degradation of OP could catalyze the depolymerization of the urethane bond in the second step and contribute to the dehydration of the polyol in the third step [26,38,47,49].

Comparing the results of T_5%_ for OP-containing materials, it can be seen that FPU-8.5 and FPU-9.0 reached the highest temperatures in the initial degradation stage (Figure 5a). At temperatures corresponding to 50% weight loss, the situation was the opposite—the highest T_50%_ value was achieved by the FPU-9.5. Comparing T_70%_ results for FPU-8.5, FPU-9.0 and FPU-9.5 materials, it can be seen that, with increasing phosphorus polyol content, these temperatures were higher and amounted to 393.2, 408.2 and 513.3 °C, respectively. OP accelerates the initial stage of degradation, but stabilizes the final decomposition of the material. Moreover, the incorporation of phosphorus into the polyurethane chain lowers the initial decomposition temperature but accelerates the char yield formation at high temperatures [23,47]. The Y values after testing in the N_2_ atmosphere for FPUs, were even three times higher compared to the reference PU and ranged from 23.91% to 24.94% (Table 4).

Thermal decomposition of reference polyurethane in an oxidative environment was more complex than an inert atmosphere (Figure 5c,d, Table 5). There were seven peaks of decomposition in the air atmosphere, which suggested an overlapping of multiple pyrolysis and oxidative reactions. The first peak in the DTG curve at about 322.2 °C (T_1max_) corresponded to the degradation of PU under oxygen influence into oligomeric compounds, low molecular weight compounds containing hydroxyl groups, CO_2_ and water. Then, the peak related to urethane bonds pyrolysis was observed at T_2max_. The peaks at T_3max_ and T_4max_ corresponded to oxidation (347.3 °C) and pyrolysis (367.3 °C) of unreacted polyol residue, respectively. At T_5max_, the oxidation of oligomeric compounds derived from PU chains decomposed with a high degradation rate. At this stage, the char, H_2_CO, CH_4_, CO, CO_2_, H_2_O and low molecular weight compounds containing –OH groups were probably formed. The next stage of degradation (T_6max_) was the pyrolysis of soft segments derived from the PU chains, during which char, compounds containing hydroxyl groups, methane and water can be formed. The last characteristic peak (T_7max_) was related to the char oxidation [50].

During the decomposition of polyurethanes containing phosphorus polyol in an oxidizing atmosphere, three stages of degradation on the DTG curves were observed (Figure 5d). The T_1max_, T_2max_ and T_3max_ temperatures corresponded to the degradation of phosphate groups, HS and SS, respectively. A similar course of the DTG curve in the air atmosphere, illustrating the degradation of phosphorus-containing PU, was presented by H. T. Q. Phan et al. [51]. The best thermal stability in N_2_/O_2_ atmosphere, among all the FPUs, was demonstrated for material, which had the highest amount of phosphorus polyol (FPU-9.5).

OP-containing polyurethanes showed, in the air atmosphere, similar to in the inert atmosphere, faster degradation in the initial stage compared to the reference material. A five percent mass loss for the REF was recorded at 307.3 °C, as well as for FPU-8.5 at 216.6 °C. It was also related to the lower thermal stability of phosphate groups in FPUs compared to ether groups in REF [52]. However, with growing temperature, the OP-containing materials were much more resistant to oxidation. Temperatures of T_50%_ were higher by about 50 °C and T_70%_ even higher by 200 °C for the FPUs, compared to the PTHF-containing material. The residual mass after the test for REF equaled to 0.00%, when for OP-containing polyurethanes R values were in the range 25.16–30.14%.

Comparing the effects of OP in the N_2_ and N_2_/O_2_ atmosphere, it can be seen that the initial stage of degradation revealed at similar temperatures (T_5%_, T_OP max_, T_1 max_). Mass losses of 50% and 70% occurred at much higher temperatures for the materials measured in the oxidizing atmosphere. After the end of the tests (at 665 °C), higher Y values for FPU-9.5 were recorded in the air atmosphere (30.14%), FPU materials were more resistant to degradation in an oxidizing atmosphere, which is significant in real conditions of high-temperature hazards.

### 3.5. The UL-94 Vertical Burning Test and LOI Results

Table 6 contains the flammability classification of all tested materials, LOI values and observations noted during UL-94 burning test. All polyurethanes containing OP were classified as V-0 according to the UL-94 standard. FPUs were characterized by the char formation during burning, which prevented spreading the flame into the material. With increasing the phosphorus content, a shorter range of flame propagation along the material was noted, especially during I flame application. Furthermore, detachment of burning pieces and appearance of dripping drops were not observed. After removing the ignition source, all PUs containing OP were extinguished within 1 s, during both the first and second flame applications.

In contrast to the FPUs, the reference material showed significantly lower flame resistance during UL-94 test performance. Flaming droplets dropped from the REF, which ignited the cotton placed on the base. For this reason, REF was classified as V-2. The burning time of this material after removing the ignition source during the first flame application was five times longer than polyurethanes containing incorporated phosphorous atoms. The char formation for PTHF-based PU was not reported.

A limited oxygen index is necessary to measure the fire spread density of a material exposed to fire in the presence of air [53]. Materials with an LOI of less than 21% are classified as highly flammable, as the air contains 21% oxygen. The higher the LOI value, the better the flame retardant properties [18]. The obtained PUs containing OP showed similar results to the LOI values, which are 3% higher than the reference material (22.4%). The presence of phosphorus polyol contributed to the improvement of the flame resistance. LOI values for OP-containing materials could probably be even better when adding suitable flame retardants to achieve a synergistic effect [54].

### 3.6. Cone Calorimeter (CC)

**Figure 6:**
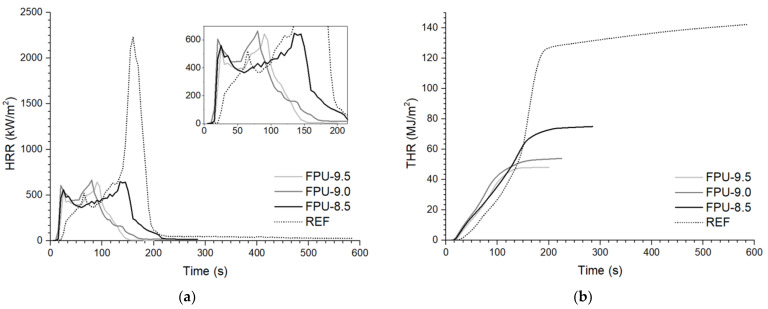
(**a**) Heat release rate and (**b**) total heat release curves for obtained polyurethanes.

HRR is one of the key parameters for assessing the combustion behaviors of materials [55]. Figure 6 shows the curves of the heat release rate (HRR) and total heat release (THR). Moreover, time to ignition (TTI), maximum heat release rate (pHRR), flame spread rate (FSR), maximum mass loss rate (pMLR), total heat release (THR), maximum average rate of heat emission (MAHRE), time to flameout (TTF), total oxygen consumed (TOC), maximum smoke production rate (pSPR), effective heat of combustion of volatiles (EHC), and residue (R) data are presented in Table 7. The pHRR value for reference PU equaled 2232 kW/m^2^. After incorporating phosphorus polyol into the polyurethane chain instead of PTHF, this value was lowered to 643–664 kW/m^2^ for FPUs. The reason for appreciable decreases in the pHRR values was the char formation on the surface of OP-containing materials. This char limited heat and mass exchange between PU materials and the flame [55], which was confirmed by the pMLR and THR results. Figure 6a shows that a relatively stable char layer was formed after two small peaks of HRR to alleviate the further FPU combustion [31]. The lowest pHRR value (643 kW/m^2^) was noted for FPU-9.5, which had the highest content of incorporated P atoms [56].

**Figure 7:**
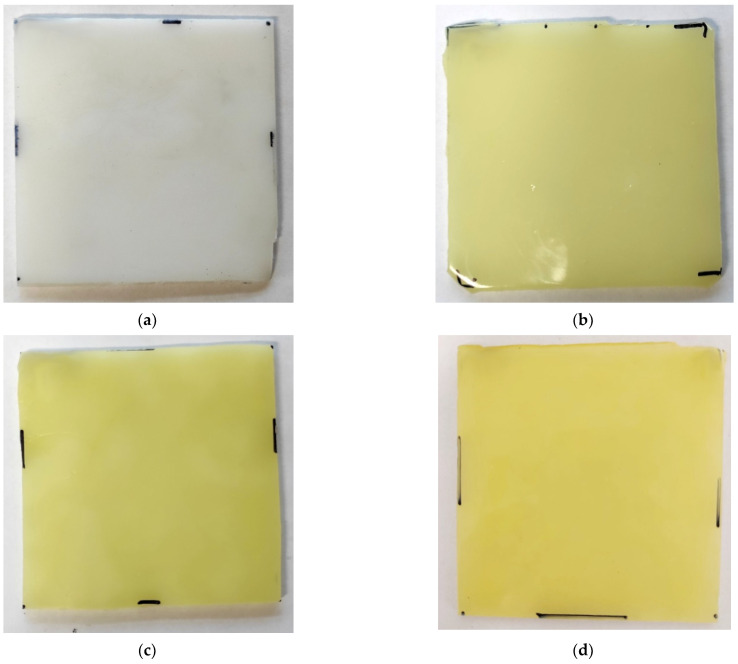
Polyurethanes before cone calorimeter tests: (**a**) REF, (**b**) FPU-8.5, (**c**) FPU-9.0, (**d**) FPU-9.5.

**Figure 8:**
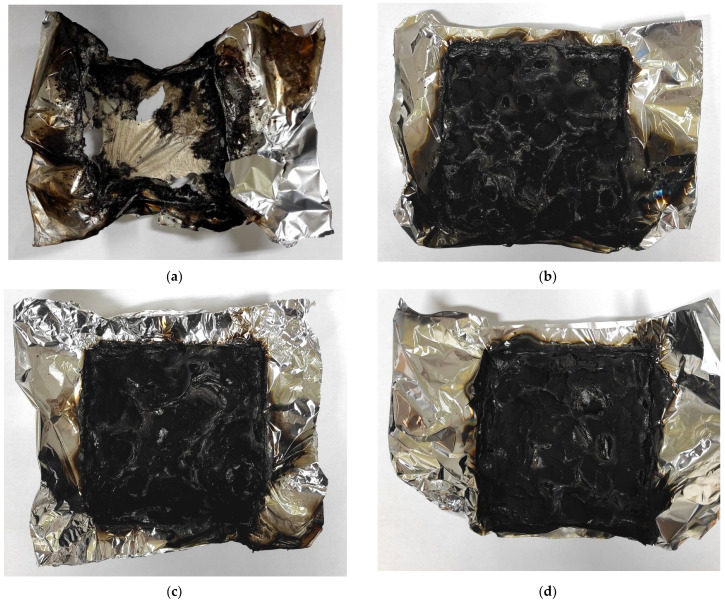
Polyurethanes after cone calorimeter tests: (**a**) REF, (**b**) FPU-8.5, (**c**) FPU-9.0, (**d**) FPU-9.5.

As mentioned in the description of the TGA tests, OP accelerates the initial stage of degradation, but stabilizes the final decomposition of the material. Time to ignition (TTI) for the reference PU was the longest and amounted to 24 s. Phosphorus flame retardants have low thermal stabilities, decompose earlier, but protect the underlying PU matrix [57]. Time to flameout (TTF) was about three times longer for REF compared to OP-containing materials and was equal to 591 s with 0.0% residue (R) after testing. The combustion of FPU-8.5, FPU-9.0, and FPU-9.5 ended after 244, 186 and 154 s, respectively, and the R values for these materials were in the range of 20.7–27.9%. Digital photographs of the materials before and after performing the cone calorimeter tests are shown in Figure 7 and Figure 8, respectively.

Flame spread rate can be calculated based on the pHRR and TTI values [31]. The lower the FSR value, the higher the fire safety of the material. For the reference PU, FSR was 93.0 kW/(m^2^s), while with an increase in the phosphorus polyol content, these results were 40.5, 44.3, and 33.8 kW/(m^2^s), respectively.

The values of total heat release (THR) decreased with the increase in the amount of phosphorus in the polyurethanes [56]. THR for REF was 132.2 MJ/m^2^, while for FPU-9.5 was equal to 47.5 MJ/m^2^. The steeper the THR curve, the faster the flame travels [58], as shown in Figure 6b.

MAHRE allows predicting fire advancement under full-scale conditions [55]. In this case, the positive effect of phosphorus polyol was also demonstrated—the value of the maximum average rate of heat emission was lowered. The result for the reference material was reduced from 677.9 to 433.7 kW/m^2^, 454.1 kW/m^2^ and 402.6 kW/m^2^ for FPU-8.5, FPU-9.0, and FPU-9.5, respectively. The values of the maximum mass loss rate (pMLR) decreased by 0.17–0.30 g/s depending on the amount of added phosphorus polyol, compared to REF.

Phosphorus atoms contribute to the durable and expansive char layer, which not only insulates the material from heat, but also from oxygen access [47,56]. The total oxygen consumed (TOC) decreased by more than 50% for OP-containing PUs compared to the reference material. Reducing the oxygen flow allows accelerating the break of the self-sustaining burning cycle [8]. The lowest TOC value equal to 36.3 g was recorded for FPU-9.5, containing the highest percentage of phosphorus.

The reduction in EHC, i.e., the effective heat of combustion of volatiles, was attributed to the gas-phase flame retardancy mechanism of OP [22,47]. This gas-phase could generate PO-type radicals and subsequently lead to flame inhibition during combustion [58]. The EHC values for the REF, FPU-8.5, FPU-9.0 and FPU-9.5 polyurethanes were 20.74, 14.01, 14.13, 12.99 MJ/kg, respectively.

Unfortunately, phosphorus flame retardants could contribute to increasing the amount of produced smoke [18,20]. The increase in the maximum smoke production rate (pSPR) for FPUs was mainly attributed to the gas-phase action of decomposed phosphorus forms, which led to incomplete combustion [31]. The lowest pSPR value among the polyurethanes including phosphorus polyol was 0.29 m^2^/s for FPU-9.0, while for REF pSPR was 0.22 m^2^/s.

### 3.7. Hardness, Density and Tensile Properties

The results of mechanical resistance, hardness and density of polyurethanes are presented in Table 8. The tensile properties have shown that, along with increasing soft segment contents, the values of the elongation at break and permanent elongation after break, follow an upward trend. The tensile strength for the materials containing OP was in the range of ca. 16 to 19 MPa. The obtained results show the deterioration of the FPUs strength properties in comparison with the reference polyurethane. Low ε values for FPUs are associated with very high Tg_SS_ values for these materials, as determined by DMTA. Nevertheless, the FPU-9.5 showed higher ε_t_ parameters compared to REF.

The PUs containing OP showed higher hardness and density compared to the REF. Large standard deviations of hardness tests results is probably the effect from insufficient heterogeneity of materials at different places caused by the use of the one-step method during synthesis [59]. The lower the amount of phosphorus polyol in the polyurethane, the lower density of the materials.

## 4. Conclusions

In this work, novel cast polyurethanes characterized by improved flame retardancy were prepared by a one-step method with the use of phosphorus-containing polyol. The FTIR spectra of materials containing OP confirm the incorporation of phosphorus polyol in the chain of obtained polyurethanes. The presence of OP in the PU structure improved the degree of phase separation (from 0.64 to 0.70 for FPUs) compared to the reference material (0.60 for REF). All prepared materials exhibited the typical XRD pattern with the most important diffraction signals at 2θ values 20.0, 19.9, 19.7 and 19.5 for REF, FPU-9.5, FPU-9.0 and FPU-8.5, respectively. The DMTA results show that better damping ability can be obtained with the higher phosphorus polyol content at the materials. Tan δ peak values for FPUs were in the range 0.24–0.39. FPUs were in a glassy state at room temperature and the storage modulus values for these materials significantly exceeded the value for the reference polyurethane. For REF, the E’_23 °C_ was 93 MPa, while for FPU-8.5, FPU-9.0 and FPU-9.5, this was 719, 927 and 1398 MPa, respectively. Thermogravimetric analysis showed that OP accelerates the initial stage of degradation but stabilizes the final decomposition of the material by char formation. T_50%_ and T_70%_ results for FPUs were better under air than nitrogen atmospheres. For example, for FPU-9.0, 50% and 70% mass losses occurred at 449.9 and 640.7 °C under air and at 288.8 and 408.2 °C under a nitrogen atmosphere, respectively. This is the opposite situation for REF mass loss and char values, which were higher under a nitrogen atmosphere. Cone calorimetry tests showed that OP worked in both the condensed and gas phases, contributing to char formation, which limited oxygen access, as well heat and mass exchange, and the production of decomposed phosphorus forms is initiated, which leads to inhibition of flame propagation during burning. The results show improved pHRR, THR, MAHRE, pMLR and EHC values compared to reference material. The maximum mass loss rate for REF was 2232 kW/m^2^ and for FPUs this was reduced to the range 643–664 kW/m^2^. The obtained results of the initial process of degradation, the behavior in the presence of oxygen and mass residues after the tests are consistent with the thermogravimetric analysis results. All materials containing OP were classified as V-0 after performing the UL-94 test for them and the LOI values were equal to 25.2–25.4%. With increasing the phosphorus polyol content, better thermal and combustion results were observed. Unfortunately, phosphorus polyol contributed to increasing the amount of produced smoke and deterioration of the FPU strength properties, which should be improved in future scientific research. This facility of the method to produce flame-retarded cast polyurethanes and presented properties makes them promising materials to encapsulate electrical and electronic elements with improved fire safety.

## 5. Patents

The method of obtaining flame-retarded polyurethanes described in this work was submitted for protection to the Patent Office of the Republic of Poland (patent application no. P.434425).

## Figures and Tables

**Table 1:** The formulations of synthesized materials.

PU Materials	PTHF (wt%)	OP (wt%)	MDI (wt%)	BDO (wt%)	Phosphorus (wt%)	Molar Ratio MDI:polyol:BDO
REF	55.59	-	36.58	7.83	0.00	1.00:0.38:0.60
FPU-8.5	-	50.81	41.36	7.83	8.63	1.00:0.45:0.53
FPU-9.0	-	53.34	39.73	6.93	9.07	1.00:0.50:0.49
FPU-9.5	-	55.87	38.15	5.98	9.50	1.00:0.54:0.44

**Table 2:** The values of hydrogen bond index (R), degree of phase separation (DPS), degree of phase mixing (DPM), and hard (HS) and soft (SS) segment contents of the polyurethanes.

PU Materials	HS (%)	SS (%)	R	DPS	DPM
REF	44.41	55.59	1.48	0.60	0.40
FPU-8.5	49.19	50.81	2.03	0.70	0.30
FPU-9.0	46.66	53.34	1.79	0.64	0.36
FPU-9.5	44.13	55.87	1.74	0.64	0.36

**Table 3:** Values of damping factor (tan δ), the glass transition temperature of the soft segments (Tg_SS_), storage modulus corresponding to this temperature (E’_SS_), storage modulus at room temperature (E’_23ᵒC_), the peak of the loss modulus (E”), the temperature range of efficient damping (∆T) and integral area tan δ peaks (S).

PU Materials	tan δ	Tg_SS_ (°C)	E’_SS_ (MPa)	E’_23°C_ (MPa)	E’’ (MPa)	∆T (°C)	S
REF	0.26	−21.2	394	93	176.6	-	13.48
FPU-8.5	0.24	25.1	624	719	248.3	-	15.74
FPU-9.0	0.33	30.5	471	927	248.3	11.46	17.00
FPU-9.5	0.39	38.4	354	1398	261.7	16.75	18.06

**Table 4:** TGA and DTG results for PU materials tested under nitrogen atmosphere.

PU Materials	T_5%_ (°C)	T_50%_ (°C)	T_70%_ (°C)	T_OP max_ (°C)	T_HS max_ (°C)	T_SS max_ (°C)	Y (%)
REF	304.7	319.7	424.7	-	319.7	422.3	7.28
FPU-8.5	218.4	285.7	393.2	246.3	293.4	310.4	23.91
FPU-9.0	220.6	288.8	408.2	246.3	294.2	308.4	24.94
FPU-9.5	212.7	297.8	513.3	246.6	329.5	358.7	24.37

**Table 5:** TGA and DTG results for PU materials tested under air atmosphere.

PU Materials	T_5%_ (°C)	T_50%_ (°C)	T_70%_ (°C)	T_1 max_ (°C)	T_2 max_ (°C)	T_3 max_ (°C)	T_4 max_ (°C)	T_5 max_ (°C)	T_6 max_ (°C)	T_7 max_ (°C)	Y (%)
REF	307.3	397.3	464.8	317.3	334.8	347.3	367.3	382.3	409.8	502.3	0.00
FPU-8.5	216.6	448.4	628.4	243.3	306.4	451.3	-	-	-	-	25.16
FPU-9.0	217.7	449.9	640.7	242.6	317.5	458.8	-	-	-	-	26.92
FPU-9.5	217.2	459.2	665.7	245.2	316.5	476.9	-	-	-	-	30.14

**Table 6:** UL-94 and LOI tests results.

PU Materials	Rating	Char Formation	The Burning Time of the Material after Removing the Fire Source		Dripping	LOI (%)
			I Flame Application	II Flame Application		
REF	V-2	no	5 s	2 s	yes	22.4
FPU-8.5	V-0	yes	1 s	1 s	no	25.4
FPU-9.0	V-0	yes	1 s	1 s	no	25.4
FPU-9.5	V-0	yes	1 s	1 s	no	25.2

**Table 7:** Cone calorimetry results, where TTI—time to ignition, pHRR—maximum heat release rate, FSR—flame spread rate, pMLR—maximum mass loss rate, THR—total heat release, MAHRE—maximum average rate of heat emission, TTF—time to flameout, TOC—total oxygen consumed, pSPR—maximum smoke production rate, EHC—effective heat of combustion of volatiles, R—residue.

PU Materials	TTI (s)	pHRR (kW/m^2^)	FSR (kW/(m^2^s))	THR (MJ/m^2^)	MAHRE (kW/m^2^)	pMLR (g/s)	TTF (s)	TOC (g)	EHC (MJ/kg)	pSPR (m^2^/s)	R (%)
REF	24	2232	93.0	132.2	677.9	0.72	591	100.2	20.74	0.22	0.0
FPU-8.5	16	648	40.5	73.8	433.7	0.55	244	56.3	14.01	0.31	26.9
FPU-9.0	15	664	44.3	52.0	454.1	0.42	186	39.9	14.13	0.29	20.7
FPU-9.5	19	643	33.8	47.5	402.6	0.47	154	36.3	12.99	0.40	27.9

**Table 8:** Tensile strength (R_m_), elongation at break (ε), permanent elongation after break (ε_t_), hardness (H) and density (ρ) of obtained polyurethanes.

PU Materials	R_m_ (MPa)	ε (%)	ε_t_ (%)	H (ShD)	ρ (g/cm^3^)
REF	36.7 ± 1.7	720 ± 5	28.1 ± 0.6	37.9 ± 3.2	1.1243 ± 0.0003
FPU-8.5	16.3 ± 1.8	57 ± 2	18.7 ± 3.7	52.3 ± 5.7	1.3267 ± 0.0006
FPU-9.0	19.6 ± 2.2	63 ± 6	24.0 ± 3.4	53.8 ± 5.5	1.3336 ± 0.0003
FPU-9.5	17.6 ± 0.6	73 ± 1	31.6 ± 3.6	45.7 ± 5.7	1.3342 ± 0.0003

## Data Availability

Data is contained within the article.
